# Lipid metabolism in type 1 diabetes mellitus: Pathogenetic and therapeutic implications

**DOI:** 10.3389/fimmu.2022.999108

**Published:** 2022-10-06

**Authors:** Jing Zhang, Yang Xiao, Jingyi Hu, Shanshan Liu, Zhiguang Zhou, Lingxiang Xie

**Affiliations:** National Clinical Research Center for Metabolic Diseases, Key Laboratory of Diabetes Immunology (Central South University), Ministry of Education, and Department of Metabolism and Endocrinology, The Second Xiangya Hospital of Central South University, Changsha, China

**Keywords:** type 1 diabetes mellitus (T1DM), dyslipidemia, β cell, inflammation, immunity

## Abstract

Type 1 diabetes mellitus (T1DM) is a chronic autoimmune disease with insulin deficiency due to pancreatic β cell destruction. Multiple independent cohort studies revealed specific lipid spectrum alterations prior to islet autoimmunity in T1DM. Except for serving as building blocks for membrane biogenesis, accumulative evidence suggests lipids and their derivatives can also modulate different biological processes in the progression of T1DM, such as inflammation responses, immune attacks, and β cell vulnerability. However, the types of lipids are huge and majority of them have been largely unexplored in T1DM. In this review, based on the lipid classification system, we summarize the clinical evidence on dyslipidemia related to T1DM and elucidate the potential mechanisms by which they participate in regulating inflammation responses, modulating lymphocyte function and influencing β cell susceptibility to apoptosis and dysfunction. This review systematically recapitulates the role and mechanisms of various lipids in T1DM, providing new therapeutic approaches for T1DM from a nutritional perspective.

## Introduction

T1DM is a chronic autoimmune disease characterized by the destroy of immune-mediated insulin-producing pancreatic β cells, which is attributed to genetic and environmental factors (1). According to the data from the International Diabetes Federation Atlas 2021, approximately 1.2 million children and adolescents are diagnosed with T1DM in 2021 (2). According to epidemiological data from high-risk areas U.S. and low-risk areas such as China, adult-onset T1DM is also more common than childhood-onset T1DM (3). Analysis of U.S. data from commercially insured individuals demonstrated the annual incidence rate of T1DM was 34.3 per 100,000 persons for ages 0–19 years and 18.6 per 100,000 persons for ages 20–64 years, but the total number of new cases in adults over a 14-year period was 19,174 compared with 13,302 in youth (4). Similarly, it has been reported that about 65.3% of the new onset T1DM cases occurred in adults as opposed to children in China (5). In addition to the increased incidence of T1DM in adults, migration studies (6), rising incidence within genetically stable populations (7), and twin studies (8) also suggest that nongenetic factors play roles in the development of T1DM. However, the environmental triggers which initiate pancreatic β cell destruction remain largely unknown.

Lipids are ubiquitously present throughout the body, which play wide and powerful roles in organisms because they participate in various basic life functions, and the abundance of lipid molecules in serum may also be closely associated with a variety of autoimmune diseases (9–11). As a pancreatic β cell-specific autoimmune disease, there is growing evidence that circulating levels of certain lipid molecules change before the onset of T1DM and even before the appearance of autoimmune antibodies in patients with T1DM (12–20) and may be associated with T1DM onset. In childhood-onset T1DM, the Finnish Type 1 Diabetes Prediction and Prevention (DIPP) study (13) showed by serum metabolomics that children who later progressed to T1DM had reduced serum levels of succinate and phosphatidylcholine at birth and the changes in these lipids were not attributable to HLA genetic risk. The metabolomic profiling in German BABYDIAB study (14) revealed that islet autoantibody-positive children had higher concentrations of odd-chain triglycerides (TGs) and polyunsaturated fatty acid (PUFA)-containing phospholipids in serum than autoantibody-negative children did. The American Diabetes Autoimmunity Study in the Young (DAISY) cohort study (21) demonstrated that plasma arachidonic acid (ARA)-derived oxylipin levels were highly associated with an increased risk of T1DM, whereas increased α-linolenic acid (ALA) and linoleic acid (LA)-related oxylipin levels were associated with a reduced risk of T1DM in children at genetic risk. The Environmental Determinants of Diabetes in the Young (TEDDY) study (18), a multinational multicenter cohort of young people with diabetes, also suggested that disturbances in lipid metabolism occur one year prior to seroconversion in children with T1DM, as evidenced by the upregulation of fatty acids, cholesterol, and phosphatidylethanolamines, and the downregulation of sphingomyelins, phosphatidylcholines, TGs, and ceramides. The abovementioned serum lipid profiles for T1DM were based on childhood onset, whereas the Swedish AMORIS cohort study (12) revealed that the serum levels of fructosamine, TGs, and apolipoprotein (apo) B were elevated 15-20 years before the onset of T1DM in adults. Given that above studies are all prospective cohort studies, so it cannot be denied that lipids are involved in the pathogenesis and progression of T1DM independently of genetic predisposition. In addition, dyslipidemia is prevalent among youth with T1DM with one third to half of youth in various studies having low-density lipoprotein cholesterol (LDL-C) above the optimal level of 2.6 mmol/L (22). A Chinese cross-sectional study based on newly diagnosed adult-onset T1DM patients from 2015 to 2017 showed that among 1158 newly diagnosed adult-onset T1DM patients, 29% had TG levels >1.7 mmol/L, 39.6% of patients had high-density lipoprotein cholesterol (HDL-C) below normal values, and more than 50.3% had (LDL-C) levels >2.6 mmol/L (23). In fact, causal relationship between lipid abnormalities and T1DM has largely not been elucidated.

Lipids function as a double-edged sword in immune response as well as inflammation. Emerging evidence demonstrates that abnormal lipid metabolism plays important roles in the pathogenesis and progression of T1DM. However, the molecular mechanisms through which lipids affect the immune-mediated β cell destruction remains unclear. In this review, we summarize the clinical and basic research evidence on specific classes of lipids in relation to T1DM with the purpose of finding possible strategies to prevent or treat T1DM from a nutritional perspective.

## Effects of lipids on T1DM

**Figure 1:**
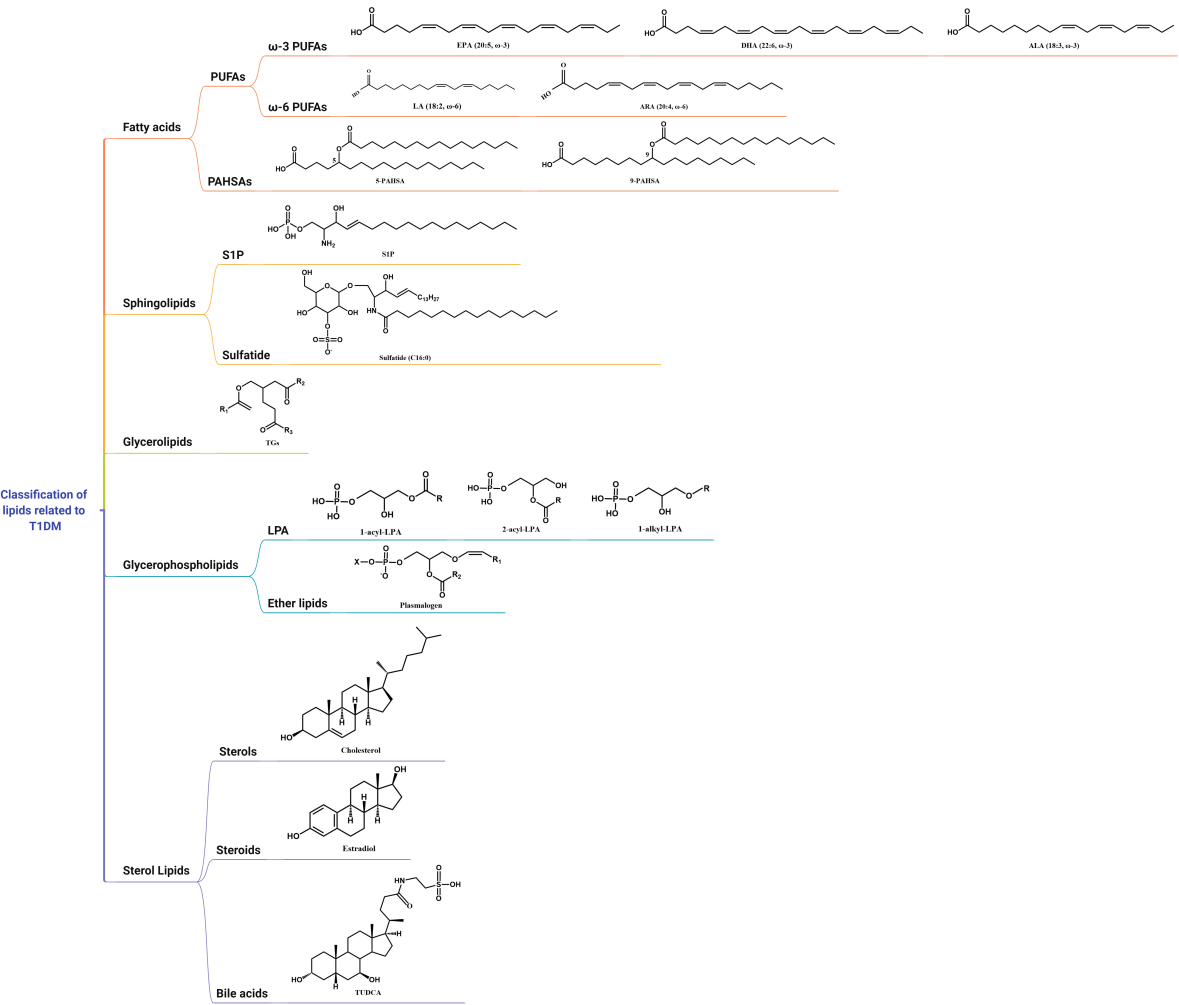
Classification and structure of lipids related to T1DM.

Lipids are mainly classified into eight categories, namely, fatty acids, sphingolipids, glycerolipids, glycerophospholipids, sterol lipids, prenol lipids, saccharolipids, and polyketides (24). There are no studies on prenol lipids, saccharolipids, and polyketides related to T1DM so far. Thus, we will discuss the association of the other five lipids with T1DM, including their possible functions and mechanisms (**Table 1**; **Figure 1**).

**Table 1:** Role of key lipids in T1DM.

Lipid	Participation in T1DM	Reference
**PUFAs**		
ω-3 PUFAs	1. Affect immunocytes differentiation and functions. 2. Restrain inflammatory signals of macrophages.	(25–27)
PAHSAs	1. Affect immunocytes activation. 2. Promote β cell regeneration. 3. Inhibit β cell ER stress.	(9)
**Sphingolipids**		
S1P	1. Mediates the targeted transport of mature immune cells. 2. Inhibits β cell apoptosis. 3. Antagonizes insulin signaling	(28, 29)
Sulfatide	1. Participates in the preservation of insulin in β cells. 2. Regulates insulin secretion.	(30–32)
**Glycerophospholipids**		
LPA	1. Mediates the targeted transport of pathogenic immune cells into islets? 2. Converts monocytes into proinflammatory macrophages?	(33, 34)
Plasmalogens	1. Scavenge intracellular reactive oxygen species and protects β cells from oxidative damage?	(13, 35)
**Sterol Lipid**		
Estradiol Bile acids	1. Promotes the production of protective cytokines by iNKT cells to prevent the development of islet inflammation and islet β cell loss. 2. Inhibits β cell apoptosis. 1. Inhibit ER stress-induced β cell apoptosis	(36, 37) (38)

### Fatty acids

Previously, free fatty acids were thought to be a metabolic hub linking obesity, insulin resistance, and type 2 diabetes (T2DM), and a recognized independent risk factor for cardiovascular disease. However, in recent years, emerging evidence shows that free fatty acids are also associated with the development of islet autoimmunity in T1DM.

#### PUFAs

PUFAs refer to straight-chain fatty acids containing two or more double bonds and having carbon chain lengths ranging from 18 to 22 carbon atoms. According to the position and function of the double bond, PUFAs are further divided into well-established ω-3 PUFAs and ω-6 PUFAs.

##### ω-3 PUFAs

ω-3 PUFAs mainly include ALA (18:3 ω-3), eicosapentaenoic acid (EPA; 20:5 ω-3), and docosahexaenoic acid (DHA; 22:6 ω-3), with ALA serving primarily as a precursor for the synthesis of EPA and DHA (39). These PUFAs are precursors to a series of bioactive lipids metabolized by cyclooxygenase (COX), lipoxygenase (LOX), and cytochrome P450s (CYPs) (40). Mammals lack the desaturase enzyme that synthesizes the relevant products and the enzyme that converts ω-6 PUFAs into ω-3 PUFAs (encoding ω-3 PUFA desaturase, fat-1), so ω-3 PUFAs can only be obtained from the dietary supply (25). The effects of ω-3 PUFAs on T1DM have been extensively studied over the past decades. Several clinical studies have shown that long-term supplementation with ω-3 PUFAs starting in infancy or childhood reduces the risk of islet autoimmunity (41–43). Indeed, higher ω-3 PUFA levels in erythrocyte membranes were also found to be associated with a reduced risk of islet autoimmunity in the DAISY study (42, 44). DHA-derived oxylipins are highly protective against T1DM (21). A recent lipidomic analysis of serum free fatty acids in childhood-onset T1DM showed that patients with T1DM may partially affect the activity of lipid elongases and thus the metabolic profile of PUFAs (45). Although supplementation with ω-3 PUFAs in infancy is a good intervention for T1D progression, a meta-analysis on the effect of ω-3 PUFAs intake in early pregnancy or early childhood on the risk of preclinical and clinical T1DM showed that supplementation with ω-3 PUFAs did not reduce the risk of T1DM (46). Additionally, a randomized, double-blind, placebo-controlled trial in adult-onset T1DM patients indicated that sustained high-dose supplementation with the ω-3 PUFAs (EPA and DHA) for up to 6 months did not improve vascular health, glucose homeostasis, or metabolic parameters in T1DM subjects (47). Compared with adult-onset T1DM, earlier nutritional intervention can significantly reduce the occurrence of T1DM in children, and even after the occurrence of diabetes, it helps to reduce the occurrence of diabetic complications, which highlights the importance and necessity of early nutritional intervention (41, 48). Therefore, to demonstrate whether supplementation with ω-3 PUFAs is effective in preventing and treating T1DM, large-scale prospective cohort studies are required including different ages and durations of disease.

**Figure 2:**
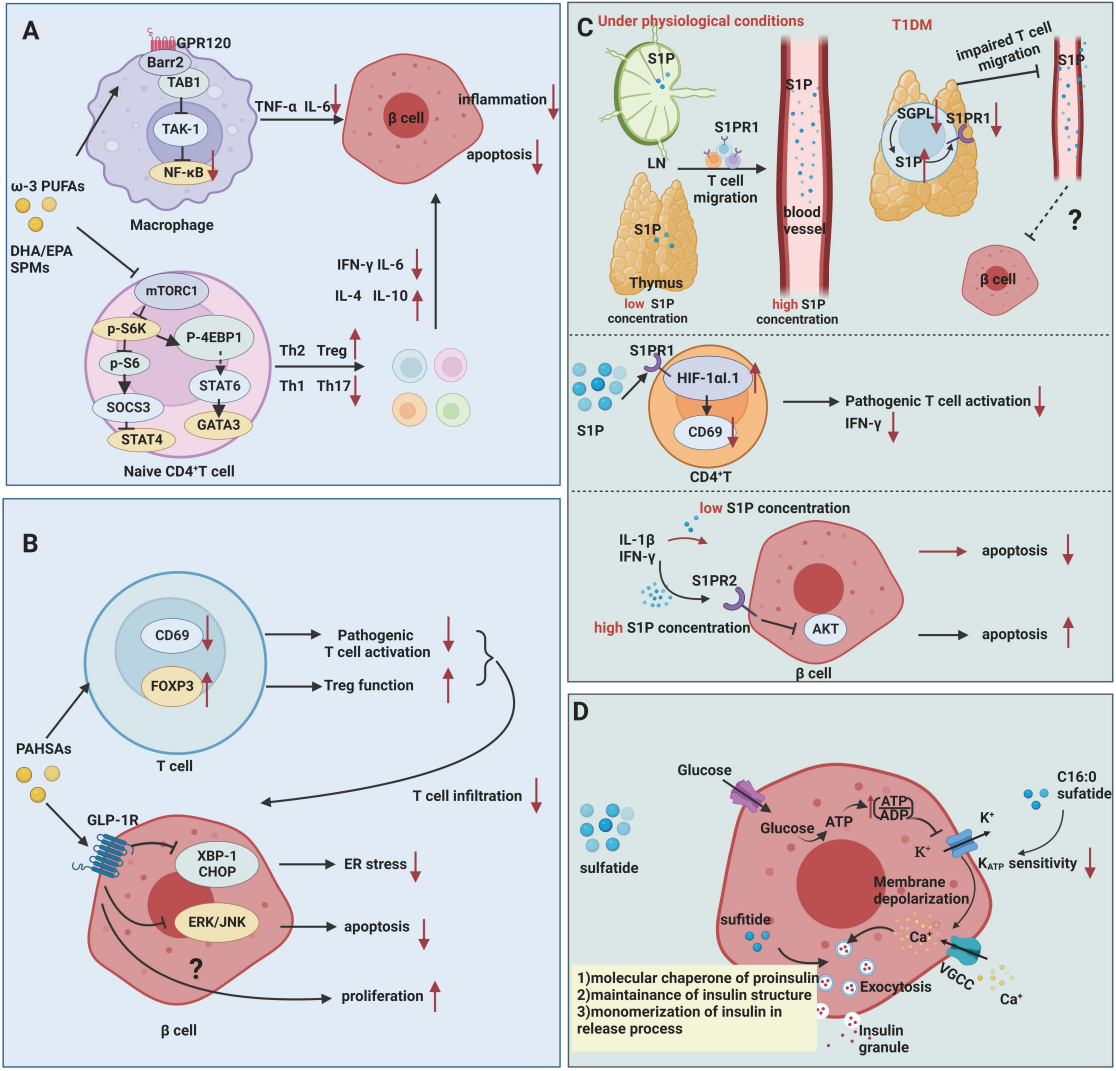
Mechanisms of fatty acids and sphingolipids in T1DM. **(A)** ω-3 PUFAs exert inhibitory effects on inflammation and autoimmunity in T1DM. ω-3 PUFAs bind to GPR120, which couples to β-arrestin2 and inhibits TAB1-induced activation of TAK1, providing a mechanism for inhibition of NF-κB related pro-inflammatory signaling pathways in T1DM. ω-3 PUFAs and their metabolites suppress the activation of Th1 cells through inhibiting of mTORC1. **(B)** PAHSAs attenuate immune responses and exert direct protective effects on β cell survival and function in T1DM. PAHSAs can reduce T cell infiltration and activation, while increasing Treg activation in the pancreas of NOD mice. PAHSAs can stimulate one of its receptors, GLP-1R, and inhibit the expression of ER stress-related protein XBP-1 and CHOP as well as apoptosis-related ERK/JNK signaling in β cell. **(C)** S1P mediates impaired mature T cell migration and activation in addition to exerting dual role in cytokine-induced apoptosis. Downregulation of degradation enzyme SGPL1 leads to high concentration of S1P in mature single positive T cell in thymus, which in turn contributing to S1PR1 ubiquitinylation and degradation. Abnormal S1P-S1PR1 signaling renders T cells in capable of migrating from thymus to blood. S1P inhibits the activation of pathogenic T cells by upregulating the HIF-1αI.1 on lymphocytes in NOD mice and downregulates CD69 and IFN-γ expression in CD4^+^ T cells. High concentration of S1P induces β cell apoptosis *via* S1PR2, while low concentration of S1P plays protective effects. **(D)** Sulfatide serves as a chaperone of proinsulin, engaging in insulin production and release. Low concentrations of sulfatide in islets may aggravate β cell destruction.

Mechanistically, ω-3 PUFAs and their bioactive derivatives mainly exert potent anti-inflammatory and immunosuppressive effects on T1DM (**Figure 2A**). At an early stage of T1DM, inflammatory mediators can induce and amplify immune attacks against pancreatic β cells while sustaining and maintaining insulitis at later stage. Therefore, the defective catabolism of inflammatory mediators and activation of persistent inflammatory signaling increase the risk of developing T1DM (49). In animal studies, Wei et al. (25) achieved endogenous production of ω-3 PUFAs by creating mfat-1 (also known as fat-1) transgenic mice that overexpress the *Caenorhabditis elegans* gene mfat-1, which encodes an ω-3 PUFA desaturase that in turn catalyzes the conversion of ω-6 PUFAs *in vivo* to ω-3 PUFAs. These mice contain higher levels of ω-3 PUFAs and lower levels of ω-6 PUFAs. In *in-vitro* experiments, the islets of mfat-1 transgenic mice were almost completely resistant to cell death induced by the proinflammatory cytokines IL-1β, TNF-α, and IFN-γ, and insulin secretion was significantly enhanced. These effects were mainly mediated by reducing prostaglandin E_2_ (PGE_2_) secretion from pancreatic β cells and inhibiting the activation of the ERK1/2 and NF-κB inflammatory pathways, which are recognized as negative regulators of insulin secretion. Jérôme et al. (50) also found that ω-3 PUFAs were enriched in pancreatic tissue in mfat-1 transgenic mice and that this transgenic mouse model was protective against streptozotocin (STZ)-induced T1DM, as evidenced by the downregulation of pro-inflammatory cytokine gene expression, the blockade of NF-κB activation, and the inhibition of ARA-derived pro-inflammatory products originating from ω-6 PUFAs. G protein-coupled receptor (GPR) 120 is a ω-3 PUFA receptor/sensor which mediates potent insulin sensitizing and antidiabetic effects *in vivo* by repressing macrophages-induced tissue inflammation (26). ω-3 PUFAs, especially DHA, lead to recruitment of β-arrestin2 to the plasma membrane and then colocalized with GPR120 followed by the GPR120/β-arrestin2 complex internalization, and finally inhibits TAK1 binding protein 1 (TAB1) activating transforming growth factor-β activated kinase 1 (TAK1), providing a mechanism for inhibition of both the NF-κB-related Toll-like receptor and TNF-α proinflammatory signaling pathways (26).

DHA-derived D-series resolvins (RvDs), protectins, and maresins (MaR) and EPA-derived E-series resolvins (RvEs) and metabolites of such bioactive lipids are also known as specialized pro-resolving mediators (SPMs), which are synthesized during the initial phase of the acute inflammatory response and have essential roles in promoting acute inflammatory regression (51). DHA-derived resolvin D1(RvD1) shows potential protection against T1DM. In the model of STZ-induced T1DM, an intraperitoneal injection of RvD1was able to increase the levels of the brain-derived neurotrophic factor with antidiabetic effects in pancreatic tissue, reduce the levels of TNF-α and IL-6 inflammatory factors in pancreatic tissue and plasma, and restore the expression of the downstream insulin signaling proteins Gsk-3β/FOXO1 and Bcl2/Pdx to promote β cell proliferation, thereby reducing the severity of STZ-induced T1DM (52).

Excessive and/or abnormal pathogenic Th1/Th17-cell responses are associated with multiple autoimmune diseases, including T1DM, and the beneficial functions of ω-3 PUFAs and their active metabolites in regulating adaptive immunity has been extensively investigated. Bi et al. (27) demonstrated that long-term dietary EPA/DHA supplementation reduced the incidence of T1DM and delayed its onset in female non-obese diabetic (NOD) mice fed an EPA/DHA-rich diet for 35 weeks beginning at 5 weeks of age. ω-3 PUFAs interfere with the differentiation of CD4^+^ T cells *via* inhibiting the mTORC1 pathway. Dietary EPA/DHA not only corrected the imbalance of effective Th1 and Th2 cells but also reduced the proportion of Th17 cells and increased the population of Tregs (27). mTORC plays a dominant role in maintaining T-cell homeostasis and determining the functional fate of cells (53). EPA/DHA and ω-6 PUFAs (particularly ARA) exert the opposite effects on mTORC1 signaling pathway. EPA/DHA reduced phosphorylation of ribosomal protein S6, thus inhibiting of mTORC1 activity. Conversely, ARA increased S6 protein phosphorylation levels by activating mTORC1 (27). Therefore, ω-3 PUFAs supplementation helps to antagonize the detrimental effects of ω-6 PUFAs and promote CD4^+^ T cell differentiation towards protective T cells.

In addition, metabolites of ω-3 PUFAs provide protective effects on the regulation of the immune system. DHA-derived RvD1, RvD2, and MaR1 reduce CD4^+^ T cell activation and inhibit the differentiation of naive CD4^+^ T cells into Th1 and Th17 cells *via* GPR32 and lipoxin receptor/N-formyl peptide receptor -2 (ALX/FPR2) while promoting the *de novo* induction of Tregs *via* GPR32 receptor (54). The EPA-derived metabolite prostaglandin D3 had a strong inhibitory effect on Th1 and Th17 cell differentiation and increased the number of Th2 and Treg cells. On the other hand, another EPA-derived metabolite, 17,18-dihydroxy-5Z,8Z,11Z,14Z-eicosatetraenoic acid (17,18-DiHETE), only reduced Th17 cell population (27). Despite the positive functions of ω-3 PUFAs metabolites in regulating immunity, no studies have been conducted to investigate the underlying mechanisms of these bioactive metabolites in the context of T1DM mice.

Overall, the successful application of ω-3 PUFAs in clinical and basic research provides hope for the prevention and treatment of T1DM, but further detailed studies on the dose of ω-3 PUFAs, the timing of supplementation, and the mechanisms of therapeutic efficacy are needed, which could help better guide the application of ω-3 PUFAs for clinical interventions in T1DM.

##### ω-6 PUFAs

ω-6 PUFAs mainly contain ARA (20:4 ω-6) and LA (18:2 ω-6). It has been shown that the anti-inflammatory effect of ω-3 PUFAs and the proinflammatory effect of ω-6 PUFAs antagonize each other, and the imbalance between ω-3 PUFAs and ω-6 PUFAs is one of the major causes of chronic diseases, such as T1DM (55), T2DM (56), and atherosclerosis (57). ARA is a precursor to a number of potent pro-inflammatory mediators including well-described prostaglandins and leukotrienes, thus ω-6 PUFAs and their derivatives are generally predisposed to promoting inflammation (58, 59). Interestingly, oxylipins derived from LA also exert some anti-inflammatory effects (21). The DAISY study detected serum oxylipins at four time points during disease progression in high-risk genotypes of children with T1DM, including 9 and 15 months of age, pre-seroconversion, post-seroconversion and diagnosis. LA-related oxylipins including 13-hydroxyoctadecadienoic acid, 9-hydroxylinoleic acid, 9-hydroxyoctadeca-10,12,15-trienoic acid, 12,13-epoxy-9-octadecenoic acid, and 9 (10)-epoxy-12Z-octadecenoic acid were inversely associated with T1DM, while 5-hydroxy-6,8,11,14-eicosatetraenoic acid, an ARA-related oxylipin, was associated with an increased risk of T1DM (21). However, a meta-analysis shows no association between ω-6 PUFAs intake and the risk of preclinical or clinical T1DM in child (46). It is widely accepted that an optimal ratio of ω-6 and ω-3 PUFAs within a healthy diet is prone to mitigating inflammation, although there is no consensus on what that ratio should be. Compared with the beneficial role of regulating inflammation of ω-3 PUFAs, ω-6 PUFAs are double-faced and still not exclusively understood.

##### Palmitic acid hydroxy stearic acids (PAHSAs)

PAHSAs are bioactive lipids with anti-inflammatory and anti-diabetic properties (**Figure 2B**). Originally discovered by the team of Barbara B. Kahn’s group (60), who performed a lipidomic analysis in adipose-selective overexpression of Glut4 mice and wild-type control mice, a series of branched fatty acid esters of hydroxy fatty acids (FAHFAs) with promising metabolic effects were identified, including PAHSAs. Unlike ω-3 PUFAs, these PAHSAs can be synthesized endogenously, and their concentration levels also correlate significantly and positively with insulin sensitivity in mice and humans (60–62). Supplementation of mice with 5-PAHSAs and 9-PAHSAs (fatty acids containing branched fatty acids on the 5^th^ and 9^th^ carbons of the fatty acid backbone) significantly improves insulin sensitivity in mice with T2DM (60). This team further reported that daily oral administration of PAHSAs in NOD mice delayed the onset of T1DM and significantly reduced the incidence of T1DM, both before and after insulitis occurrence (9). In terms of immune regulation, PAHSAs reduced T cell and B cell infiltration and CD4^+^ and CD8^+^T cell activation, while increasing Treg activation in the pancreas of NOD mice (9). PAHSAs can also attenuate cytokine-induced β cell death and increase β cell viability. The protective functions were attributable to PAHSAs-mediated reduction in endoplasmic reticulum (ER) stress and extracellular signal regulated protein kinase (ERK)/c-Jun N-terminal kinase (JNK) activation in pancreatic β cells, which appeared to be mediated in part by glucagon-like peptide 1 (GLP-1) receptor (9). Currently, the scientific exploration of therapeutic strategies for T1DM has been mainly focused on two aspects: immunotherapy to suppress or remove autoimmune cell damage to β cells (1, 63), and stem cell therapy to promote β cell regeneration and repair (64, 65). PAHSAs, as emerging lipids, have unexpected beneficial effects in regulating autoimmune responsive T cell and islet β cell regeneration, but whether the results of rodent experiments can be applied to humans requires more clinical evidence. Recently, Barbara B. Kahn’s group has identified adipose triglyceride lipase (ATGL) as a candidate for FAHFA biosynthesis *in vivo* (66). Understanding how ATGL production and activity are regulated could be a promising strategy to increase levels of these beneficial lipids in the context of T1DM.

### Sphingolipids

Sphingolipids are prevalent components of eukaryotic membranes, and metabolites of sphingolipids, including sphingosine-1-phosphate (S1P) and sulfatide, are signaling molecules that regulate a variety of cellular processes. Several clinical studies have revealed changes in serum sphingolipid profiles before and after the development of autoimmunity in T1DM (67–69).

#### S1P

S1P is a multifunctional bioactive sphingolipid secreted into circulation mainly by erythrocytes and vascular endothelial cells through sphingosine kinase 1 (SPHK1) or SPHK2 (10). S1P in lymph fluid primarily derived from lymphatic endothelial cells (70). It can bind to five G protein-coupled receptors (S1P receptors, S1PRs) and be involved in the pathogenesis of multiple sclerosis, rheumatoid arthritis, systemic lupus erythematosus and other autoimmune diseases (71). S1P remains at low concentrations in tissues, lymphoid organs as well as thymus and at high concentrations in blood (10). Maintenance of extracellular concentration gradient depends on by degradative enzymes such as phospholipid phosphatase 3 (LPP3) (72) and S1P lyase 1 (SGPL1) (73). Although animal studies and cellular studies describe the linkage between S1P and T1DM, no exact data on S1P concentration in the pancreas of T1DM patients and no detailed data on S1P receptor, transporter protein, and metabolic enzyme expression in the pancreas are available. Explorations of S1P have mainly focused on animal studies and cellular studies. However, dynamic changes in plasma and local tissue S1P have not been recorded in animal models of autoimmune development of T1DM.

Current studies suggest that S1P may be involved in T1DM by mechanisms including 1) involvement in impaired lymphocyte migration and activation and 2) extracellular S1P protection of β cells from cytokine-mediated apoptosis and dysfunction (**Figure 2C**).

Studies have shown that S1P is involved in impaired lymphocyte trafficking and lymphocyte activation. S1PR1 is expressed on the lymphocytes, and S1P-S1PR1 axis mediates lymphocyte migration from lymph nodes to blood and lymph fluid depending on gradient concentrations of S1P (74). Specifically, chemokine receptor CC-chemokine receptor 7 (CCR7) expressed on the naïve and central memory T cells mediates lymphocytes entry and retention in lymph node. S1P-S1PR1 axis facilitates T cells egress by countering retention signals mediated by CCR7 (75, 76). In NOD mice, the perivascular spaces of the thymus are filled with mature CD4^+^T, CD8^+^T, and Foxp3^+^ Treg cells. The decreased expression of S1PR1 and degradation enzyme SGPL1 were observed in these cells. Diminished expression of SGPL can lead to a higher concentration of S1P in local surroundings in thymus (28), which is able to induce S1PR1 internalization, followed by ubiquitinylation and degradation of S1PR1 (77). Thus, mature immune cells in thymus fail to response to S1P gradient-mediated migration and are detained in thymus in NOD mice. S1PR1 expression and S1P/S1PR1 interactions are partially involved in the impaired T-cell migration in the pathogenesis of T1DM in NOD mice. After all, migration-related molecules including alpha 5 beta 1 integrin (VLA-5, a fibronectin receptor), alpha 6 beta 1 integrin (VLA-6, a laminin receptor), CXCR4 and CXCL12 also participate in impaired thymocytes migration in the context of NOD mice, which are beyond the scope of this article (78). Besides, *in vivo* studies have shown that S1P inhibits the activation of pathogenic T cells by upregulating the hypoxia-inducible factor-1 alpha short isoform I.1 (HIF-1αI.1) on lymphocytes in NOD mice and downregulates CD69 and IFN-γ expression in CD4^+^ T cells (79). However, S1P-S1PR1 signaling reportedly increased Th17 cell differentiation from CD4^+^T cells and suppressed Treg cell numbers and functions in other autoimmune diseases (10). It is unclear whether the net effect of S1P signaling in T1DM is detrimental or protective. Thus, clarifying the actual changes in sphingolipid metabolites during T1DM progression and developing selectively targeting drugs to control S1P production, transportation and degradation may be a promising way to treat autoimmune diseases such as T1DM.

It was shown that low extracellular concentrations (<5 μM) of S1P significantly reduced cytokine-induced apoptosis in rat primary pancreatic β cells and INS-1 cells, a kind of rat β cell line (29). The levels of TUNEL staining, cytochrome C, and caspase-3 in proinflammatory factor-stimulated β cells were reduced after treatment with nanomolar concentrations of S1P. However, high extracellular concentrations (>5 μM) of S1P are toxic to insulin-secreting cells, leading to the activation of caspase-3 and NF-κB (29). This was confirmed by the findings of Japtok et al. (80). In Lewis rats pancreatic β cells, elevated extracellular S1P concentrations were found to antagonize insulin-mediated β cell growth and survival *via* S1PR2 and then to inhibit AKT signaling. Besides, the reduction of intracellular S1P concentration by an overexpression of S1P lyase in insulin-secreting INS1E cells is able to inhibit the cytokine-induced reduction in cell proliferation and activation of caspase-3 (29), which suggests that the effects of exogenously added and intracellularly produced S1P on β cells depend mainly on the local concentration of S1P and the duration of exposure to diabetogenic conditions (81). To determine whether S1P is able to protect β cells in T1DM conditions and protect pancreatic β cells, further validation is needed in animal models of autoimmune diabetes as well as in the human population.

#### Sulfatide

Sulfatide is a kind of glycosphingolipid originally identified in neural tissue and later in islets of Langerhans’ cells. The pancreas contains large amounts of short-chain (16:0) sulfatide, and in the pancreas of some species, 50% of all glycosphingolipids are of this type (69). Buschard et al. initially identified autoimmune antibodies against sulfatide in the serum of newly diagnosed T1DM patients, suggesting that sulfatide induces an immune response during the natural progression of T1DM. This group (68) also found reduced expression of enzymes related to sphingolipid metabolism and decreased levels of sulfatide in the islets of newly diagnosed T1DM patients. This suggests that the decrease in sulfatide promotes β cell damage in T1DM. Animal studies have shown that autoantibody reactivity to sulfatide in NOD mice increases with age (82). Fenofibrate is a classical anti-lipid drug that helps reduce LDL-C, triacylglycerol, and cholesterol levels in the blood and has emerged as a promising therapeutic strategy in T1DM due to its activation of sulfatide biosynthesis in islets (68). Supplementation with fenofibrate for 32 weeks prior to the onset of insulitis in NOD mice leads to pancreatic lipidome remodeling and increased levels of very long-chain sphingolipids and completely prevents the development of T1DM (68, 83). Mechanistically, intracellular sulfatide functions as a chaperone of insulin, participating in the preservation of insulin crystals in β cells and promoting the instantaneous monomerization of insulin during extracellular action (30, 84) (**Figure 2D**). In addition, the metabolism of glucose in β cells generates ATP, and the elevated ATP/ADP ratio leads to the closure of ATP-sensitive potassium channels (K_ATP_) and membrane depolarization, which in turn activates voltage-gated Ca2^+^ channels to promote the release of insulin granules. In contrast, in *in-vitro* experiments, extra β cell C16:0 sulfide lipids inhibit glucose-stimulated insulin secretion by decreasing the sensitivity of K_ATP_ to ATP, mediating alternating rest in β cells (31, 32, 69), which play an important role in the first phase of insulin secretion. The reduced levels of pancreatic sulfatide may contribute to the loss of first-stage insulin secretion during the development of T1DM (85). The ability to increase islet sulfatide levels in NOD mice to attenuate islet inflammation and reduce the incidence of T1DM suggests that regulating sulfatide production may be a promising lipid target for the treatment of T1DM, but more clinical and basic research is needed to prove its efficacy.

### Glycerolipids

TGs are usually considered to be correlated with atherosclerosis (86). However, growing evidence indicates that TGs also play indispensable roles in the pathogenesis of T1DM. The Finnish DIPP study showed reduced levels of TGs in pediatric patients with T1DM before seroconversion to positive islet autoantibodies during follow-up. The TEDDY study (18) went further by identifying a lipid phenotype associated with the progression of T1DM and the onset of islet autoimmunity in children, identifying a phenotype including a decrease in TG levels. However, a prospective study of adult-onset T1DM found elevated serum TG levels 15-20 years prior to diagnosis (12). Insulin increases lipoprotein lipase activity and degrades TGs, which can explain the elevated TGs due to insulin deficiency (87, 88). In addition, TGs and apoB/apoA-I levels are thought to be associated with insulin resistance. T1DM patients also have insulin resistance, especially those using insulin (89). Thus, elevated lipid levels may contribute to the development of adult-onset T1DM by impairing insulin sensitivity, whereas the levels of TGs in childhood-onset T1DM may be less damaging to islet β cells.

### Glycerophospholipids

Glycerophospholipids, which are composed of a glycerol backbone, two molecules of fatty acids, and one molecule of phospholipids, are the most abundant class of phospholipids in the organism. In addition to being a major component of cell membranes, they are also involved in a variety of cellular activities and organismal physiological activities as signal transduction molecules. Both animal studies and clinical studies have shown that perturbations of glycerophospholipids are associated with the pathogenesis of diabetes (90).

#### Lysophosphatidic acid (LPA)

LPA is by far the smallest and structurally simplest glycerophospholipid and the smallest biologically active lipid. LPA is synthesized *via* the extracellular and intracellular pathways, with the intracellular pathway acting only as an intermediate product of glycerophospholipid metabolism, whereas LPA produced by the extracellular pathway exerts a wide range of biological effects through its interaction with six G protein-coupled receptors (LPAR1-6) (91, 92). Autotaxin (ATX, gene name Enpp2), one of the most extensively studied LPA signaling pathway-related enzymes, is proposed to be the primary contributor of extracellular LPA, with lysophospholipase D activity and the ability to convert lysophospholipids to LPA (92). It is well established that the ATX-LPA signaling axis is associated with rheumatoid arthritis (93), psoriasis (94), multiple sclerosis (95), and several other autoimmune diseases.

**Figure 3:**
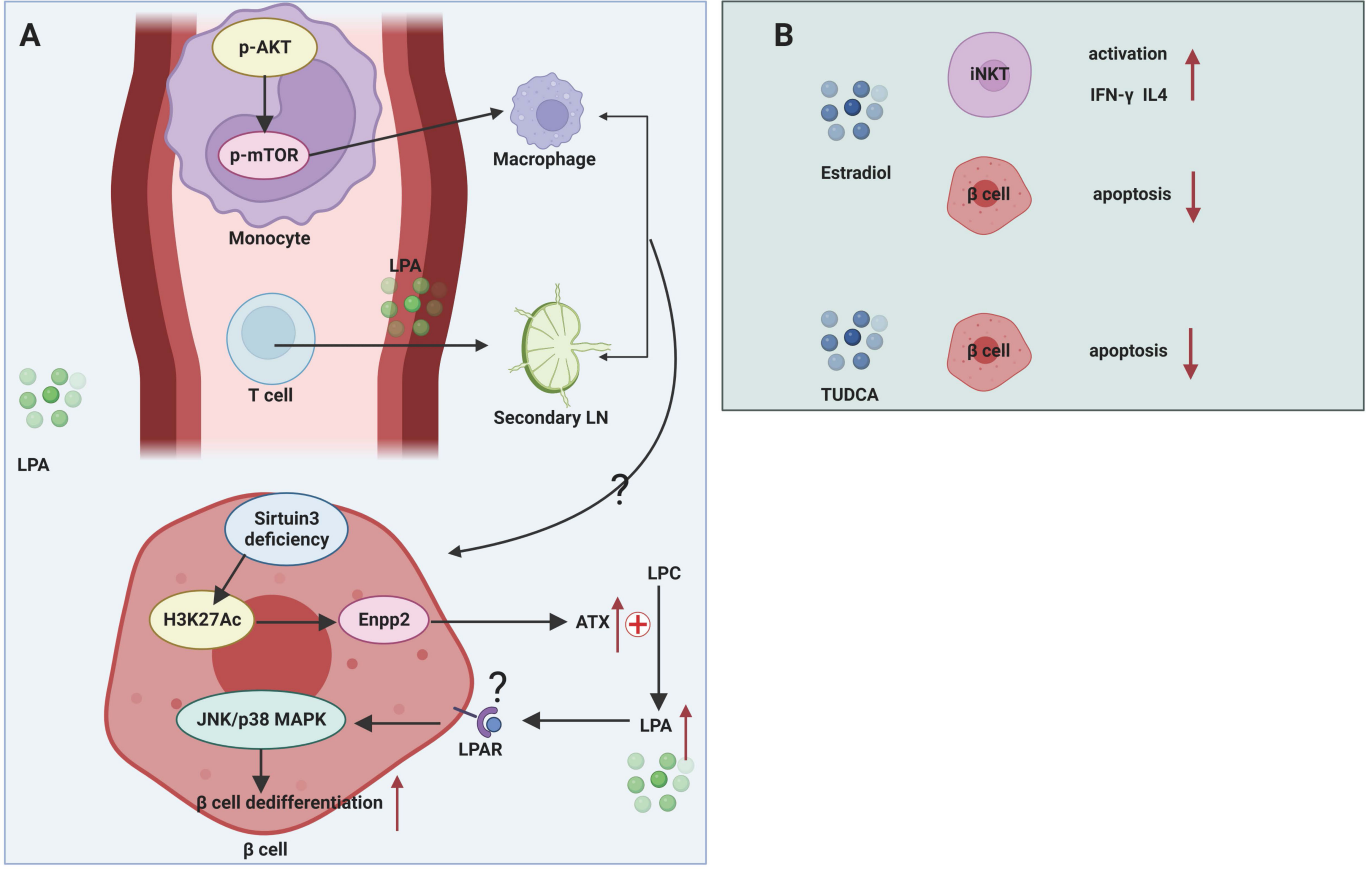
Mechanisms of glycerophospholipids and sterol lipids in T1DM. **(A)** LPA participates in pathogenic T cell migration from blood to secondary LNs. LPA can transform murine monocytes to macrophages by stimulating AKT/mTORC pathway. Sirtuin 3 deficiency of β cell facilitates the transcription of Enpp2 and subsequent high levels of ATX in extracellular environment. ATX converts LPC to LPA, which activates JNK/p38 MAPK pathway and facilitates β cell dedifferentiation and dysfunction. **(B)** Estradiol has a protective role in β cells by activating iNKT cells and mitigating β cell apoptosis. TUDCA can alleviate ER stress.

The role of LPA in maintaining β cell function is controversial (**Figure 3A**). The effect of LPA on glucose metabolism was first reported by Yea et al. (96), who found that LPA was able to lower blood glucose levels in normal and STZ-induced T1DM mice without affecting insulin levels, and this effect was blocked by the LPAR1/3 blocker Ki16425. However, in high-fat-fed obese mice, contradictory results were obtained, with both exogenous and endogenous LPA capable of inhibiting insulin secretion from β cells and affecting glucose homeostasis (97). This effect may act by suppressing GLP-1 secretion from intestinal L cells, which in turn impairs glucose-stimulated insulin secretion (98). Recent study also demonstrated that sirtuin 3 knockdown in β cell induced increased expression of acetyl- Lys 27 of histone H3 (H3K27Ac) and signal transducer and activator of transcription (STAT) 5, which can bind to the promoter region of the Enpp2 gene facilitating the transcription of ATX. Activated ATX-LPA signaling induced phosphorylation of JNK1/2 and p38 MAPK and consequently lead to β cell dedifferentiation and β cell dysfunction (99). The relatively contradictory results in mouse models may be attributed to the diversity of LPA isoforms, widely overlapping expression patterns in multiple tissues, and the effects of LPA acting on multiple downstream pathways mediated by different LPARs. Therefore, more intensive studies should be of great help to clarify the function of specific LPA isoforms in T1DM.

The ATX-LPA axis mediates the migration and differentiation of immune cells. The ATX-LPA axis differs from S1P in that LPA has been shown to mediate the entry of lymphocytes from the blood into secondary lymphoid organs. ATX is abundantly expressed in the high endothelial vein (HEV), which controls the movement of lymphocytes from the blood into lymphoid organs (100). This suggests that during the progression of T1DM, the ATX-LPA axis may mediate the infiltration of pathogenic immune cells from the circulation into the pancreatic draining lymph nodes and then into the pancreatic tissue, resulting in autoimmune damage to the islets. In addition, LPA has been reported to induce the conversion of human monocytes into macrophages, and the Akt/mTOR pathway and the pathway represented by PPARγ play key roles in LPA-induced macrophage formation and differentiation (33). Macrophages are an important component of innate immunity and are responsible for the autoimmune initiation and pathogenic phase of T1DM (101). It suggests that LPA may be involved in the macrophage-mediated development of T1DM.

#### Ether lipids

Ether lipids are a unique class of glycerophospholipids whose alkyl chains are attached to the sn-1 carbon atom of glycerophospholipids by ether bonds, accounting for approximately 20% of the total mammalian phospholipid pool. Of these, plasmalogens are the most abundant ether lipids in serum (35). Plasmalogens connect fatty alcohols containing vinyl-ether bonds at the sn-1 carbon atom of the glycerol backbone and PUFAs at the sn-2 carbon atom (35). The Finnish DIPP cohort study noted that acetal ether lipids were decreased in the serum of children subsequently diagnosed with T1DM (13). However, there is currently no conclusive evidence as to whether the decreased ether lipid levels and the progression of T1DM are related. Normal pancreatic β cells can produce as many as 1 million insulin molecules per minute and produce a 50-fold insulin secretory response to hyperglycemia. Highly loaded β cells are therefore also susceptible to environmental stimuli including oxidative stress and ER stress (101, 102). Plasmalogens are cellular antioxidants containing vinyl unsaturated groups that scavenge a variety of intracellular reactive oxygen species (ROS) and protect cellular unsaturated membrane lipids from damage by singlet ROS (35). Furthermore, due to abundant levels of PUFAs at the sn-2 position, plasmalogens are also considered key storage depots of PUFAs. These PUFAs can be cleaved and metabolized into potent second messenger molecules, such as protectins and resolvins, to induce anti-inflammatory and anti-apoptotic effects (103). Whether increasing the level of plasmalogens in β cells can protect pancreatic β cells from oxidative damage requires further investigation.

### Sterol lipids

Sterol lipids, exemplified by sterols, steroids, bile acids, and derivatives, are reportedly associated not only with T1DM-related complications but also with T1DM disease progression.

#### Sterols

Cholesterol is the most abundant sterol in plasma, and cholesterol is closely related to lipoprotein classes. Apo A and apo B are important components of HDL-C and LDL-C, respectively. Given that patients with T1DM have an increased risk of cardiovascular disease and mortality (104), most of the current studies in the field of diabetes tend to focus on the effects of cholesterol and its related apolipoproteins on diabetic cardiovascular disease. However, the effects of cholesterol and its related apolipoproteins on T1DM are gaining attention. It has been shown that HDL-C has the ability to regulate glucose metabolism in diabetes and that infusion of HDL-C in diabetic individuals improves pancreatic β cell function, possibly due to reduced apoptosis (105). A recent cohort study showed that the apo A-I of adult-onset T1DM was negatively associated with the risk of T1DM and that the apo B/apo A-I was positively associated with the incidence of T1DM (12).

#### Steroids

Steroids include estrogens, androgens, progestogens, glucocorticoids, and mineralocorticoids. Among them, estradiol (E2) shows a pivotal role in T1DM than previously appreciated. Different from rheumatoid arthritis, multiple sclerosis, and Crohn’s disease, T1DM is the only common autoimmune disease that does not exhibit a female predominance, which is evidenced by male predominance of T1DM in Caucasians (ratio M/F: 1.7) (106) and especially male bias after the age of 15 years (ratio M/F:1.8) (107). The pubertal period is in a close proximity to decreased incidence of T1DM in girls who preserve stronger residual β-cell function than boys (108). This indicates the possible protective function of estradiol on T1DM. Congruent with the phenomenon, a cross-sectional study revealed that adolescents with T1DM had lower levels of serum oestrogenic activity (109). However, to date no large cohort study has demonstrated the causal role of estrogen in the pathogenesis of T1DM.

The functionality of the immune system exhibits a remarkable sexual dimorphism. Generally, females manifest more robust immune response than males (110). Estrogens elicit huge impact on regulating innate immunity and adaptive immunity in the normal immune system and in different diseases such as tumor and autoimmune disease. Subcutaneous implantation of estrogen extended-release pellets in NOD mice, both before and after the onset of insulitis, was able to prevent the development of T1DM by enhancing the production of IFN-γ and IL-4 by iNKT cells (36) (**Figure 3B**). Although this report shows a protective effect of estrogens in NOD mice, no further study has been done on how estrogens affect the activation and function of iNKT cells in the context of NOD mice. This study does not rule out beneficial effects of estrogens on other immune cells. After all, estrogens were reportedly involved in macrophage polarization by skewing macrophages towards an ant-inflammatory M2-like phenotype (111, 112). Additionally, compelling evidence demonstrates that estrogens can promote Treg differentiation and increase the immunosuppressive activities in order to restore immune tolerance. Low levels of E2 enhance Th1 response while elevated E2 favor a Th2 response (110). However, beneficial effects of estrogen regulating other immune cells have not yet been demonstrated in humans as well as in NOD mice.

It is well established that E2 improves β cell function and viability and protects β cell from proinflammatory cytokine-induced apoptosis, oxidative stress and glucolipotoxicity (113). High levels of E2 (pregnancy period) showed improvement in insulin sensitivity and glucose-stimulated insulin secretion (GSIS) in female New Zealand obese mice (114). E2 protects β cells against different apoptotic insults such as STZ and proinflammatory cytokines, and this effect is mediated in part through three estrogen receptors (ERα, ERβ and G protein-coupled estrogen receptor) (37). Considering the systemic nature of estrogenic effects and the multifaceted nature of intracellular hormonal signaling, long-term estrogen administration is undesirable in young newly diagnosed T1DM patients because of the adverse effects on the reproductive system that are associated with long-term estrogen administration. Therefore, it is important to conduct further studies to determine the pathways and mechanisms involved in how sex hormones affecting immune system and β cell apoptosis to develop selective estrogen receptor modulators.

#### Bile acids

Bile acids have been extensively studied for their beneficial roles in regulating glucose tolerance, insulin sensitivity and energy metabolism *via* nuclear Farnesoid X receptor and Takeda G protein-coupled receptor 5 (115). Despite promising discoveries on the regulation of immune cells by bile acids in the context of inflammatory bowel disease, the role of bile acids in T1DM is largely unknown. A prospective cohort study in Norway quantified bile acids in umbilical cord blood of child who later developed T1DM aiming to seek for possible metabolites that contribute to the prediction of T1DM (116). However, no bile acids show their potential association with T1DM (116). In addition, a small cross-sectional study also showed that serum BA levels were not associated with the pathogenesis of latent autoimmune diabetes in adults (117). Clinical studies may be of heterogeneity with respect to sample handling, measurement method, exposures measurement, results report, statistical analysis, and endpoint design, which may cover up the function of bile acids in T1DM. Taurine-conjugated ursodeoxycholic acid (TUDCA) administration in NOD mice during the pre-diabetic stages protected β cells against ER stress-induced apoptosis in an activating transcription factor-6 (ATF6)-dependent manner, while TUDCA treatment did not affect related immune cells population implicated in T1DM (38) (**Figure 3B**). Cinnamaldehyde, a type of aldehyde in the bark of *Cinnamomum trees*, interfered with host gut microbiota and affected bile acid pools *in vivo*, thereby regulating the expression levels of genes involved in glucose metabolism and reducing blood glucose levels in STZ-induced T1DM mice (118). However, this study did not perform in-depth mechanistic studies of specific bile acid-mediated protection in NOD mice.

## Conclusion and perspective

In this review, we have discussed in detail how lipids modulate immune response, inflammation response and influence β cell function in the context of T1DM. Overall, ω-3 PUFAs and their derivatives, PAHSAs, S1P, estrogen exert protective effects in T1DM by regulating immune cell activation, differentiation, migration, and cytokine production in T1DM. Meanwhile, ω-3 PUFAs play a pivotal role in anti-inflammation *via* inhibition of NF-κB-mediated signaling. PAHSAs, S1P, estrogen, and bile acids also contribute to attenuating β cell apoptosis and improving β cell viability. Sulfatide serves as chaperon of insulin to promote insulin preservation and secretion.

Research on the pathogenesis of T1DM has focused on immune damage to islets by pathogenic immune cells. Increasing evidence suggests that changes in lipid levels in plasma and local tissue occur prior to the onset of T1DM pathogenesis and even prior to seroconversion of islet autoimmune antibodies, indicating that early metabolic disturbances may be an earlier response relative to autoimmunity and that identification of these pre-autoimmune metabolic alterations may contribute to the study of disease pathogenesis, making lipid profiles a potential biomarker for detecting disease severity and extending the intervention window for T1DM prevention. Taking nutritional intervention for example, nutrition affects all physiological processes including those linked to the development and function of the immune system (119). Early specific lipids intake such as fish oil (rich in ω-3PUFAs) can enhance protective immunity while also limiting aberrant inflammatory responses in T1DM. Therefore, we should take nutritional intervention into consideration to promote the development of rational diet-based therapeutic options for the prevention and treatment of disease.

In addition, several T1DM lipidomic studies have focused on infants and children, without special attention to the characteristics of lipid metabolism in the development of adult-onset T1DM and more studies should be endeavored in the future to explore the differences in the pathogenesis of adult-onset T1DM and childhood-onset T1DM to better exploit the role of individualized medicine and precision medicine. After all, the epidemiological data from both high-risk areas of T1DM in Northern Europe and low-risk areas in China show that adult-onset T1DM is more common than childhood-onset T1DM (3). lipids and their metabolites are involved in T1DM by regulating immunity and inflammation. However, there are still many unresolved issues, and future research on lipids in T1DM may need to be conducted in the following areas: 1) Lipid signaling is subject to complex and sophisticated regulation and is associated with immune cell development and differentiation, transportation, and subtype functions. Given the prevalence of lipid perturbations in organisms, the regulation of lipid signaling for the immune system may be systemic and thus requires the development of targeted modulation of local immunity for the prevention and treatment of T1DM. 2) Because of the complexity of lipids at the biochemical level and the diversity of isoforms, the identification and characterization of proteins and enzymes involved in the downstream roles of active lipid production, transportation, and receptors are key to understanding their physiological roles, and these proteins and enzymes may also be novel lipid signaling candidates for targeted therapy. 3) How lipids mediate immune cell and pancreatic islet β cell crosstalk and the exact molecular and immunologic mechanism are still worthy of further study.

## Author contributions

JZ was mainly responsible for designing the content structure, collecting literature, and writing the original draft. YX made suggestions for designing and writing the original manuscript. JH provided editing assistance and computer support. SL and ZZ made useful suggestions for revising this manuscript. LX supervised the whole work, performed analysis, editing, validation and made great contributions in re-designing the work and revising the manuscript. All authors contributed to the article and approved the submitted version.

## Funding

This work was supported by the National Key Research and Development Project (2018YFE0114500 to YX), the Science and Technology Innovation Program of Hunan Province (2021RC 3033 to YX), the Natural Science Foundation of Hunan Province for Youths (2022JJ40718 to LX, 2022JJ40689 to JH), the Natural Science Foundation of Changsha (kq2202404 to JH).

## Conflict of interest

The authors declare that the research was conducted in the absence of any commercial or financial relationships that could be construed as a potential conflict of interest.

## Publisher’s note

All claims expressed in this article are solely those of the authors and do not necessarily represent those of their affiliated organizations, or those of the publisher, the editors and the reviewers. Any product that may be evaluated in this article, or claim that may be made by its manufacturer, is not guaranteed or endorsed by the publisher.
